# B- and T-lymphocyte number and function in HIV^+^/HIV^−^ lymphoma patients treated with high-dose chemotherapy and autologous bone marrow transplantation

**DOI:** 10.1038/srep37995

**Published:** 2016-12-01

**Authors:** Diego Bertoli, Alessandro Re, Marco Chiarini, Alessandra Sottini, Federico Serana, Viviana Giustini, Aldo M. Roccaro, Chiara Cattaneo, Luigi Caimi, Giuseppe Rossi, Luisa Imberti

**Affiliations:** 1 Centro di Ricerca Emato-oncologica AIL (CREA), ASST Spedali Civili, Brescia, Italy; 2 Hematology, ASST Spedali Civili, Brescia, Italy

## Abstract

Combination of anti-retroviral therapy, high-dose chemotherapy (HCT) and autologous stem cell transplantation (ASCT) has led to an improved survival of HIV^+^ non-Hodgkin lymphoma (NHL) patients. We compared T- and B-cell subset recovery and related capability to respond to *in-vitro* stimulation, as well as T-cell repertoire modifications of HIV^+^ and HIV^−^ NHL patients undergoing HCT and ASCT as first-line consolidation or salvage treatment, using sequential blood samples obtained before and at 3, 6, 12 and 24 months after ASCT. B lymphocyte recovery occurred earlier, reaching higher levels in HIV^+^ patients as compared to HIV^−^ patients and healthy controls; in particular, immature and naïve B cells were significantly higher in HIV^+^ patients who had received rituximab in the pre-ASCT period. These lymphocytes equally responded to *in-vitro* stimulation. Newly produced T cells similarly increased in HIV^+^ and HIV^−^ NHL patients, but their levels remained constantly lower than in healthy controls. T lymphocytes showed a reduced proliferative capacity, but their repertoire was reassorted by the treatment. The functional and numeric B-cell recovery and the qualitative modifications of T-cell receptor repertoire may explain, at least in part, the success of this aggressive therapeutic approach in HIV^+^ patients.

The introduction of combination anti-retroviral therapy (cART) has modified the natural history of HIV infection, reducing HIV-related morbidity and mortality, a significant portion of which, however, is still accounted for by HIV-associated lymphoma^1^. Moreover, immune preservation with cART has changed the therapeutic approach to HIV-associated lymphoma, allowing the use of aggressive treatment strategies, including high-dose chemotherapy (HDC) with autologous stem cell transplantation (ASCT). This approach has been explored at several Institutions in patients with refractory or relapsed HIV-associated lymphomas, showing high clinical efficacy with low toxicity and lack of significant increase in opportunistic infections^2^,^3^,^4^,^5^,^6^,^7^,^8^,^9^. ASCT has also been used with encouraging results as early consolidation treatment after first-line therapy in HIV^+^ patients with lymphoma at high risk of relapse^2^,^10^. The effects of ASCT, used as salvage treatment, were similar between HIV^+^ and HIV^−^ subjects, and a trend towards a lower probability of relapse after ASCT was observed in HIV^+^ patients^10^,^11^,^12^. The initial concerns related to the possibility that HDC could exacerbate the immune depression already present in HIV^+^ patients, leading to infection progression, were ruled out by the demonstration that ASCT does not enhance viral replication or the peripheral HIV reservoir in the long term and does not worsen the T-cell impairment^13^. Rather, a T-cell recovery has been described, probably related to the maintained thymus capability of transplanted patients to generate new T cells, as demonstrated by the peripheral increase of lymphocytes containing T-cell receptor excision circles (TRECs)^+^ cells^13^,^14^. The post-ASCT immune recovery appears not to be different in HIV^+^ versus HIV^−^ patients because total and naïve CD4^+^-lymphocytes, as well as TRECs, are similarly increased in both groups of patients^15^. This suggests that conditioning regimens may create an identically appropriate lymphoid niche that can be equally replenished by the transferred cells in both groups of patients^15^,^16^. While it has been reported that the lymphocyte recovery also involves CD8^+^ and CD19^+^ cells, which undergo a rapid expansion in both HIV^+^ and HIV^−^ groups after the period of aplasia^15^, whether the kinetics of the recovery of CD4^+^, CD8^+^ and CD19^+^ cells and their subsets differ among HIV^+^ and HIV^−^ patients remain not fully answered. Moreover, it is not known whether lymphocytes that replenish the immune system in the post-ASCT period are functional. Finally, whether the T-cell receptor (TCR) repertoire undergoes similar modifications in HIV^+^ and HIV^−^ patients has not been explored yet.

## Results

### Patients’ characteristics and treatment

Of the 32 enrolled patients (17 HIV^+^ and 15 HIV^−^), 20 (11 HIV^+^ and 9 HIV^−^) were included in the analysis. Twelve patients were not analyzed for immune recovery either because they relapsed early after ASCT (4 in each group) or because we included in the study only patients whose samples were collected at least at four different time points. At study entry, all HIV^+^ patients were receiving cART; median time from HIV diagnosis to cART initiation and from HIV diagnosis to ASCT was 30 (range: 5–192) and 46 (6–336) months, respectively.

The main characteristics of the patients and clinical data are shown in Table 1. The prevalence of men in the HIV^+^ group reflects the epidemiology of HIV infection in Italy^17^, while the difference in the lymphoma histology reflects the different epidemiology of NHL in HIV^+^ and HIV^−^ populations^18^. This translates into a different percentage of patients treated in the pre-ASCT period, with the anti CD20 monoclonal antibody rituximab, which was administered only in patients with CD20^+^ lymphoma. Induction regimens for HIV^+^ patients included cyclophosphamide, doxorubicin, vincristine, and prednisone (CHOP) +/− rituximab (n: 8); high dose methotrexate-containing regimens (n: 2); and doxorubicin, cyclophosphamide, vincristine, bleomycin, prednisone, and etoposide (VACOP-B; n: 1). Platinum-based regimens were administered to 4 of these patients as salvage therapy. Induction regimens for HIV^−^ patients included CHOP and rituximab (n: 7); intensified CHOP and rituximab (n: 2). As for HIV^+^, also two HIV^−^ patients received platinum-based regimens as salvage therapy.

The number of mobilized CD34^+^ cells were significantly lower in HIV^+^ compared to HIV^−^ patients, but similar in rituximab versus non-rituximab-treated HIV^+^ patients. The observed differences in the number of mobilized CD34^+^ cells could be explained on the basis of: (1) heterogeneity in the therapy used for stem cell mobilization between HIV^+^ and HIV^−^ patients; (2) different CD34^+^ mobilization goal^19^ (3) the depletion of hematopoietic reservoir in HIV^+^ patients^20^,^21^. However, the number of infused CD34^+^ cells was comparable and not statistically different between the two groups of patients.

Patients received antibacterial, antifungal and antiviral prophylaxis until stable engraftment, while trimethoprim/sulfamethoxazole prophylaxis was suspended after stem cell infusion. None of the HIV^+^ patients interrupted cART due to oral mucositis or other toxicities, nor modified the cART due to viral response failure. A single intravenous immunoglobulin injection has been administered to one HIV^+^ and 3 HIV^−^ patients. Of note, this was given before engraftment, few days after transplant. A patient with detectable HIV viremia at ASCT became negative early afterwards, while 4 had a short-lasting detectable viremia after ASCT. Before engraftment, 4 HIV^+^ and 2 HIV^−^ patients had a bacterial documented infection, and 2 HIV^+^ patients had CMV reactivation. During the 2 years observation period, 2 herpes zoster infections and 2 CMV reactivations were seen in the HIV^+^ group, and 1 episode of pulmonary infection happened in both groups of patients.

### Quantitative post-ASCT immune recovery

**Figure 1:**
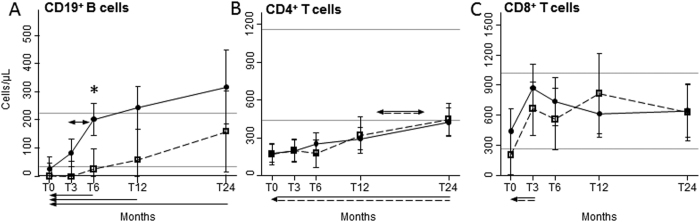
B- and T-cell recovery. Mean number of peripheral CD19^+^ (**A**), CD4^+^ (**B**), and CD8^+^ (**C**) lymphocytes at the indicated time points in HIV^+^ (black dots, solid lines) and HIV^−^ (empty squares, dashed lines) patients. Error bars represent the 95% confidence interval of the mean. Grey horizontal lines represents the reference range, defined as the highest and lowest values found in HC. Arrows made by double (dashed and solid) lines indicate significant ANOVA main effects, without significant interaction, as follows: horizontal double-headed arrows indicate significant differences between the indicated time-points; single-headed arrows indicate significant differences between the indicated time-points and T0. When interaction is significant, post-hoc comparisons are indicated as follows: * significant differences between HIV^+^ and HIV^−^ patients at the indicated time point; double-headed arrows, solid or dashed: significant variation between adjacent means within HIV^+^ or HIV^−^ patients, respectively; single-headed arrows, solid or dashed: significant variation in respect to T0 within HIV^+^ or HIV^−^ patients, respectively. T0: before ASCT (baseline); T3, T6, T12, and T24: at 3, 6, 12, and 24 months after ASCT, respectively.

Immediately after ASCT, total CD19^+^ B lymphocytes of HIV^+^ and HIV^−^ patients were below the lower values observed in healthy controls (HC), but differentially increased after ASCT, being significantly and generally higher in HIV^+^ patients, going beyond the highest value found in HC after 12 and 24 months (Fig. 1A). Specifically, B-cell number started to increase at 3 months post-ASCT and doubled in the following 3 months only in HIV^+^ patients, leading to significant differences between the two groups after 6 months. The mean values of B cells of HIV^−^ patients returned within the normal range at 12 and 24 months after ASCT. Post-ASCT median values of serum IgG increased preferentially in HIV^+^ patients (from 654 [range: 338–1680] mg/dL at 3 months since ASCT to 983 [623–1670] mg/dL at 24 months) that in HIV^−^ subjects (from 621 [160–1312] mg/dL to 691 [224–1313] mg/dL).

Before ASCT, the number of CD4^+^ T lymphocytes of HIV^+^ and HIV^−^ patients were significantly lower as compared to HC, but significantly and comparably increased from 12 to 24 months after ASCT (Fig. 1B). The mean CD8^+^ cell counts were below the lowest values of HC at the pre-ASCT time point in HIV^+^ individuals only, significantly increased after 3 months in both groups, and always remained within the reference interval (Fig. 1C).

### Quantitative post-ASCT B-cell subset recovery

**Figure 2:**
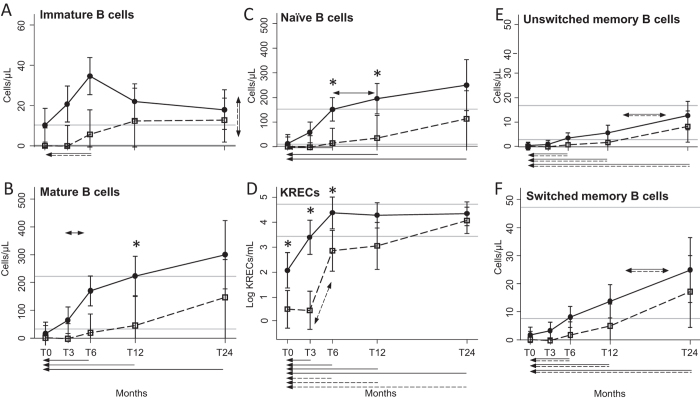
B-cell subset recovery. Number of peripheral B-lymphocyte subsets at the indicated time points in HIV^+^ (black dots) and HIV^−^ (white squares) patients. Immature B cells (**A**) are CD19^+^CD10^+^ lymphocytes, mature B cells (**B**) are CD19^+^CD10^−^ lymphocytes. Mature B cells can be divided into IgD^+^CD27^−^ naïve B cells (**C**), IgD^+^CD27^+^ unswitched memory B cells (**E**), and IgD^−^CD27^+^ switched memory B cells (**F**). The number of KRECs is given as log/mL (**D**). Error bars represent the 95% confidence interval of the mean. Grey horizontal lines represent the reference range defined as the highest and lowest values found in HC. Arrows made by double (dashed and solid) lines indicate significant ANOVA main effects, without significant interaction, as follows: horizontal double-headed arrows indicate significant differences between the indicated time-points; single-headed arrows indicate significant differences between the indicated time-point and T0. When interaction is significant, post-hoc comparisons are indicated as follows: * significant differences between HIV^+^ and HIV^−^ patients at the indicated time point; double-headed arrows, solid or dashed: significant variation between adjacent means within HIV^+^ or HIV^−^ patients, respectively; single-headed arrows, solid or dashed: significant variation in respect to T0 within HIV^+^ or HIV^−^ patients, respectively. T0: before ASCT (baseline), T3, T6, T12, and T24: at 3, 6, 12, and 24 months after ASCT, respectively.

The better recovery of total B cells of HIV^+^ individuals involved immature CD19^+^CD10^+^ cells, which increase was significantly higher but not at a specific time point (Fig. 2A), and mature CD19^+^CD10^−^ B cells (Fig. 2B), that grew faster in HIV^+^ patients starting from 3 months, and becoming significantly higher than in HIV^−^ patients at 12 months after ASCT. The most represented mature B-cell subset, namely the IgD^+^CD27^−^ naïve population, showed a significant expansion in HIV^+^ patients compared to HIV^−^ patients at 6 and 12 months after transplantation (Fig. 2C). The early and preferential expansion of these B-lymphocyte subsets in HIV^+^ patients was confirmed by the analysis of K-deleting recombination excision circles (KRECs), which are the products of B-cell receptor rearrangements and therefore markers of B-cell neo-production. In HIV^+^ patients, KRECs were significantly higher already at baseline and increased as soon as 3 months after ASCT, while in HIV^−^ patients the increase was slightly delayed. At 24 months after ASCT, KRECs become similar in the two groups, and also those of HIV^−^ patients finally moved into the normal range (Fig. 2D). No significant differences were detected between the two groups of patients for both CD19^+^ CD10^−^IgD^+^CD27^+^ un-switched and CD19^+^CD10^−^IgD^−^CD27^+^ switched memory subsets (Fig. 2E and F).

**Figure 3:**
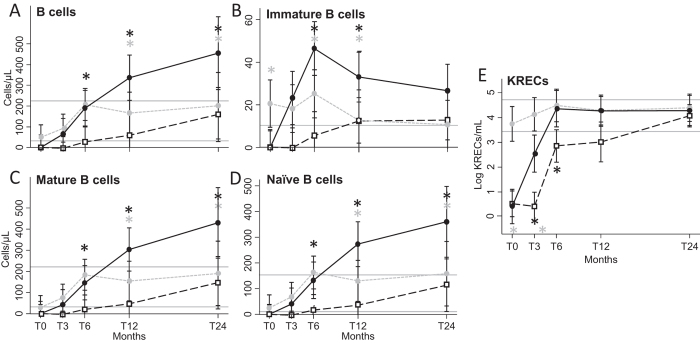
Number of peripheral B-cell subsets and KRECs. B-cell subsets and KRECs were evaluated at the indicated time points in HIV^+^ patients that have received (black solid lines and dots) or not (grey dashed lines and dots) adjuvant rituximab, and in HIV^−^ patients (black dashed lines and clear squares), who have all received rituximab. B cells (**A**) are CD19^+^ lymphocytes, immature B cells (**B**) are CD19^+^CD10^+^ lymphocytes, mature B cells (**C**) are CD19^+^CD10^−^ lymphocytes, and naïve B cells are IgD^+^CD27^−^CD19^+^CD10^−^ lymphocytes (**D**). The number of KRECs is given as log/mL (**E**). Grey horizontal lines represent reference range defined as the highest and lowest values found in HC. *Significant differences obtained by planned contrasts between cell subsets of HIV^+^ patients who received rituximab in comparison to HIV^−^ patients (black asterisks) or to HIV^+^ patients who did not receive rituximab (grey asterisks) at the indicated time points. T0: before ASCT (baseline), T3, T6, T12, and T24: at 3, 6, 12, and 24 months after ASCT, respectively.

B-cell subset values of rituximab-treated HIV^+^ and HIV^−^ patients with diffuse large B-cell lymphoma (DLBCL), were compared with those of rituximab-untrated HIV^+^ patients with other lymphoma histologic types. We found that rituximab strongly influenced the levels of B-cell subpopulations and KREC production in HIV^+^ patients, both before and after ASCT (Fig. 3). In addition, we found that complete or partial remission status did not exert any effect on B-cell recovery in HIV^+^ patients (data not shown).

### Functional post-ASCT B-cell recovery

**Figure 4:**
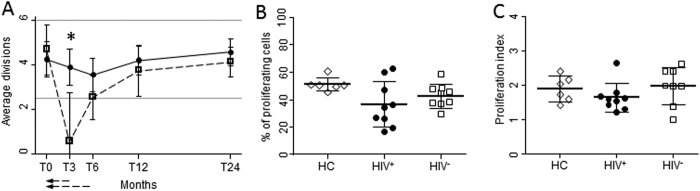
B-cell functional response. The average number of *in-vivo* B-cell divisions (**A**), the percentage of cells in the final culture that divided at least once (**B**) and the average number of divisions after *in-vitro* stimulation (**C**) were evaluated in HIV^+^ (black dots) and HIV^−^ patients (white squares) at the indicated time points and compared with that of HC (rhombi). Error bars represent the 95% confidence interval of the mean. Grey horizontal lines represent the highest and lowest values measured in HC. Two HIV^+^ and two HIV^−^ patients were not analyzed for functional study for the lack of cells at the baseline time point. *Significant differences between HIV^+^ patients in comparison to HIV^−^ patients at the indicated time-point. Single-headed dashed arrows: significant variation in respect to T0 in HIV^−^ patients. HC: healthy controls; T0: before ASCT (baseline), T3, T6, T12, and T24: at 3, 6, 12, and 24 months after ASCT, respectively.

*In-vivo* replication history of B lymphocytes, evaluated by measuring the average number of their divisions, was within the normal range in both HIV^+^ and HIV^−^ patients (Fig. 4A). The only exception was documented within HIV^−^ patient samples collected at 3 months post ASCT, where the number of B-cell divisions fell below the minimum value of HC, thus resulting significantly lower than that of HIV^+^ individuals, likely reflecting the lack of B cells early after ASCT. B-cell functional response was evaluated by CFSE assay, measuring the ability of patient cells to proliferate after combined stimulation with anti-Ig for B-cell receptor cross-linking and CpG, which targets toll-like receptor 9, in presence of interleukin-10, known to increases mRNA expression of toll-like receptor 9^22^, and interleukin-2, which sustains B-cell proliferation^23^. The fraction of B cells that divided at least once (Fig. 4B) and the average number of divisions performed by responding cells (Fig. 4C) did not differ significantly between HIV^+^ and HIV^−^ patients and HC, indicating that B lymphocytes displayed an *in-vitro* capability to proliferate after stimulation.

### Quantitative post-ASCT T-cell subset recovery

**Figure 5:**
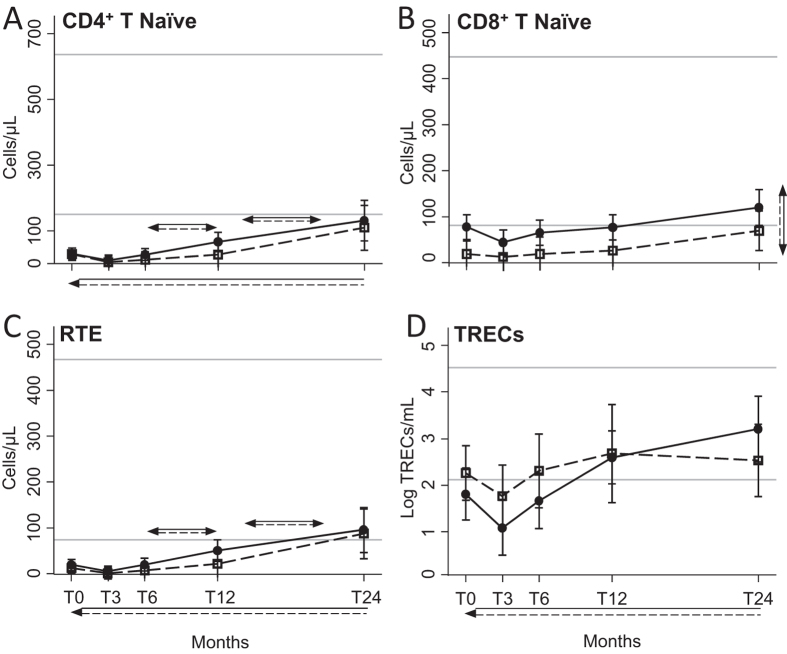
Number of peripheral CD4 and CD8 lymphocyte subsets. CD4 and CD8 lymphocytes were evaluated at the indicated time points in HIV^+^ (black dots) and HIV^−^ (white squares) patients. The presence of CD45RA and CCR7 allowed us to identify CD45RA^+^CCR7^+^ CD4^+^ (**A**) and CD8^+^ (**B**) naïve lymphocytes. RTE were naïve CD4^+^ lymphocytes expressing the CD31 molecule (**C**). The number of TRECs was measured by real-time PCR and is given as log/mL (**D**). Error bars represent the 95% confidence interval of the mean. Grey horizontal lines represent the reference range, defined as the highest and lowest values found in HC. Arrows made by double (dashed and solid) lines indicate significant ANOVA main effects, without significant interaction, as follows: horizontal double-headed arrows indicate significant differences between the indicated time-points; vertical double-headed arrows indicate a difference between HIV^+^ and HIV^−^ patients, not depending on time point; single-headed arrows indicate significant differences between the indicated time-point and T0. RTE: recent thymic emigrants; T0: before ASCT (baseline), T3, T6, T12, and T24: at 3, 6, 12, and 24 months after ASCT, respectively.

Naïve CD4 (Fig. 5A), recent thymic emigrants (RTE; Fig. 5C) and TRECs^+^ (Fig. 5D) lymphocytes similarly and significantly increased starting from 6 months after ASCT in HIV^+^ and HIV^−^ patients. Of note, naïve CD4 cells never reached the lower limit of the reference range found in HC. In contrast, the overall level of naïve CD8^+^ cells was significantly higher in HIV^+^ patients, where we observed that cells went above the lowest value obtained in HC after 12 months since ASCT (Fig. 5B). In both groups of patients, CD4^+^ T_CM_ significantly increased in respect to the baseline, and especially starting from 12 months of therapy, but the values were always under those of HC (Supplementary Figure 1A). On the contrary, CD4^+^ T_EM_ increased in the first 12 months of therapy (Supplementary Figure 1B). The number of CD8^+^ T_CM_ was significantly higher in HIV^+^ patients, with values always close or higher than the top values found in HC (Supplementary Figure 1C), while CD8^+^ T_CM_ similarly increased only in the months immediately after ASCT (Supplementary Figure 1D).

### Functional post-ASCT T-cell recovery

**Figure 6:**
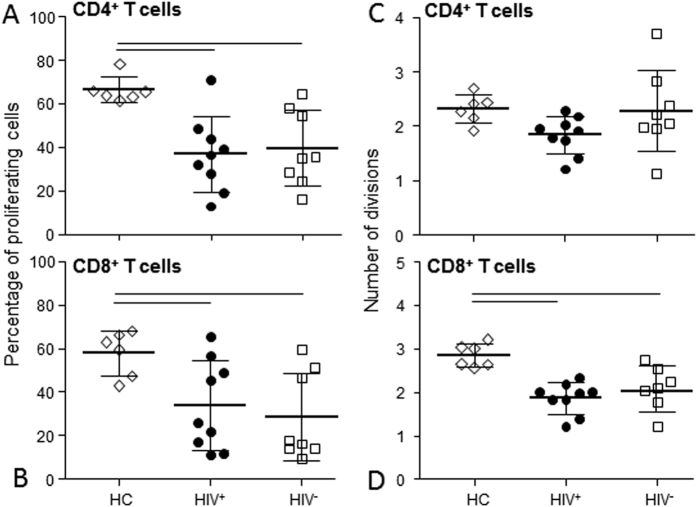
T-cell functional response. The percentage of CD4^+^ (**A**) and CD8^+^ (**B**) cells that divided at least once and the average number of divisions of CD4^+^ (**C**) and CD8^+^ (**D**) lymphocytes after *in-vitro* stimulation with PHA were evaluated in HIV^+^ (black dots) and HIV^−^ (white squares) patients at the indicated time points and compared with that of HC (rhombus). Two HIV^+^ and two HIV^−^ patients were not analyzed for functional studies due to the lack of cells at the basal time point. One data point for CD8^+^ cells divisions in HIV^+^ patients was considered a significant outlier and removed. Horizontal lines indicated significant differences between cell subsets of HIV^+^ patients and HIV^−^ patients in comparison to control. HC: healthy controls; T0: before ASCT (baseline), T3, T6, T12, and T24: at 3, 6, 12, and 24 months after ASCT months after ASCT, respectively.

The capability of T cells to respond to *in-vitro* stimulation was assessed by CFSE assay after stimulation with PHA. The percentage of CD4^+^ and CD8^+^ cells that divided at least once after 4 days of culture was significantly lower in HIV^+^ and HIV^−^ patients compared to HC (Fig. 6A and B). The average number of CD4^+^ cell divisions upon stimulation was similarly modulated in both patients and HC (Fig. 6C), while CD8^+^ cell divisions were reduced compared to HC (Fig. 6D).

### TCR repertoire analysis

**Figure 7:**
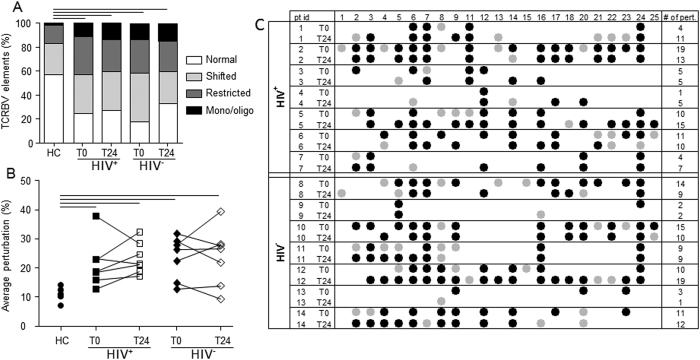
TCR repertoire analysis. (**A**) Percentage of normally distributed, shifted, restricted, and mono/oligoclonally expanded TCRBV elements. Displayed bar lengths were calculated as the group proposed categories. The reported significance was obtained comparing the within-patient proportions of pooled shifted, restricted and mono/oligoclonal vs normal TCRBV chains between HIV^+^ and HIV^−^ patients by analysis of variance. (**B**) Average percentages of TCRBV perturbations in HC, and in HIV^+^ and HIV^−^ patients at the indicated time points. Each dot represents the global average perturbation of the TRCBV repertoire in one subject. Horizontal lines indicate significant differences between cell subsets of HIV^+^ and HIV^−^ patients in comparison to HC. (**C**) Map representing the complementarity-determining region 3 (CDR3) distribution perturbation at the single-TCRBV, single-patient level. Black and grey dots represent the TCRBV families whose perturbations are respectively higher than the mean + 3 SD and mean + 2 SD of the value seen in the corresponding TCRBV family in HC. The number of these over-perturbed TCRBV elements is indicated in right column. HC: healthy controls; pt id: patient identification; TCRBV: T-cell receptor variable beta chain; T0: before ASCT; T24: 24 months after ASCT.

TCR diversity was evaluated before and 24 months after ASCT in 14 (7 HIV^+^ and 7 HIV^−^) patients. No correlation was found between the extent of therapy-induced lymphopenia and the degree of repertoire perturbation observed at the pre-ASCT period (data not shown). The mean proportion of TCRBV elements with normal, shifted, restricted and mono/oligoclonal profiles was significantly higher in samples obtained from the two groups of patients both in pre- and post-ASCT periods in comparison to samples of HC (Fig. 7A). Accordingly, the mean percentages of TCRBV perturbations were significantly higher in HIV^+^ and HIV^−^ patients at both time points (Fig. 7B). However, if TCRBV perturbations were evaluated at single-TCRBV chain and single-patient level, the types of TCR repertoire restrictions appeared different because certain perturbations of TCRBV families found in the pre-ASCT period were lost 24 months after ASCT. In parallel, for other TCRBV families, certain over-perturbations became evident only after the treatment (Fig. 7C and Supplementary Figure 2A). Finally, an unsupervised hierarchical clustering of the average perturbation changes, calculated as fold-change between post- and pre-ASCT periods, could group 6 out of the 7 HIV^+^ patients and 6 out of the 7 HIV^−^ patients (Supplementary Figure 2B).

## Discussion

We found that the profound depletion of the B-cell population observed in HIV^+^ and HIV^−^ NHL transplanted patients at the pre-ASCT time point was followed by a differential and long lasting expansion of naïve and immature B cells that progressively and significantly increased in HIV^+^ patients to overcome the values observed in HC. While there were no differences in B-cell functionality between the two groups, HIV^+^ patients also showed a better trend towards an IgG increase after ASCT in comparison to HIV^−^ patients. The observed differences in the B-cell compartment between HIV^+^ and HIV^−^ patients, treated or not with rituximab before ASCT, are intriguing. Previous results obtained in HIV^−^ subjects with NHL demonstrated that an anti-CD20-based treatment induces a complete depletion of B lymphocytes, followed by a delayed recovery of memory B cells with abnormal function, which leads to a significantly increased incidence of hypogammaglobulinemia, lasting several months after ASCT^24^,^25^. However, we found that non-rituximab-treated non-DLBCL HIV^+^ patients showed a pattern of B-cell subset increase closer to that of HIV^−^ rituximab treated DLBCL patients, while the highest number of B-cell increase was preferentially observed in HIV^+^ rituximab treated DLBCL patients. Possibly, the diverse B-cell increase in the two groups of HIV^+^ and HIV^−^ patients could be related to the different molecular characteristics, cell origin, and prognosis of DLBCL, which are known to be related to a different incidence of EBV infection^26^. Indeed, in the pre-cART and pre-rituximab era, this infection was detected in 63% of HIV^+^, but only in 3% of HIV^−^ patients. Nevertheless, similar studies have not been performed in patients treated with cART; moreover, EBV was measured only in a minority of our patients, so we cannot reach any conclusion on the role of EBV in mediating differential B-cell recovery in our HIV^+^ patients.

Previous data on T-cell recovery obtained by measuring TRECs content in HIV^+^ patients have been criticized because the results may be altered by the influence of ongoing HIV replication on the rates of cell division, apoptosis, and life span of CD4 cells^27^,^28^. This was not the case of our patients because they had a good control of HIV infection as result of the cART responsible for a reduced cell activation, proliferation, and apoptosis^29^,^30^. In addition, both approaches used to quantify thymic output, namely TREC quantification and RTE phenotyping, confirmed that the kinetics of increase of newly produced CD4^+^ lymphocytes is not different in our HIV^+^ and HIV^−^ patients. However, although increased, these cells always remained at low levels, and the percentage of CD4^+^ lymphocytes that responded to stimulation was lower than in HC in both groups. While CD4^+^ T-cell recovery following ASCT has been previously characterized, at least partially, in HIV^+^ patients, potential fluctuations of CD8^+^ cells are less defined. It has been shown that HIV infection does not influence naïve CD8^+^ T-cell levels in lymphoma patients with and without HIV infection candidates for ASCT^31^, but the kinetic measurements and a phenotypic characterization of CD8 subsets were not previously reported. Phenotypic characterization of CD8^+^ T-cell-recovery among HIV^−^ lymphoma patients undergoing ASCT has suggested a preferential expansion of antigen-primed CD8^+^ cells rather than CD8^+^ naïve cells^32^. Our findings show that immune recovery preferentially involved CD8 T_CM_, as well as naïve CD8^+^ cells. Moreover, naïve CD8^+^ cells were more expanded in HIV^+^ rather than in HIV^−^ patients, across all the evaluated time points, starting from the pre-conditioning (T0), going to the 24 months of therapy (T24). The percentage of CD8^+^ lymphocytes that proliferated upon stimulation was significantly lower in all patients as compared to HC. Therefore, CD8^+^ cells are functionally different from those of HC, and may represent a population of “exhausted” clonal T cells, similar to those found during physiological aging^33^,^34^,^35^,^36^. Accordingly, as observed in the elderly, and despite the observed increase in newly produced diversified T cells in both groups of patients, TCR repertoires at 24 months after ASCT are as restricted as in the pre-transplant period. However, we could also demonstrate relevant therapy-induced modifications of TCRBV chain profiles, including enlargement in TCR heterogeneity of T cells expressing certain TCRBV, and contraction in diversity of cells bearing other TCRBV, as well as disappearance of some clonal expansions and appearance of others. Therefore, our data differ from those reporting that patients undergoing ASCT after HDC regenerate clonal expansions consistent with those found in the pre-treatment period^37^. Furthermore, these modifications could be different in HIV^+^ and HIV^−^ patients as shown by unsupervised hierarchical clustering discriminating between the two groups. Finally, TCRBV modifications are not merely due to a physiologic repertoire “drift” over time because TCR repertoire of HC is extremely stable^38^.

Taken together these findings indicate that T- and B-cell recovery following HDC and ASCT is similar or even better in HIV^+^ than in HIV^−^ patients. Thus, the functional and numeric B-cell recovery and the qualitative modifications of T-cell repertoire may explain, at least in part, the success of this aggressive therapeutic approach in HIV^+^ patients, both considering the low number of infectious complications commonly seen in these patients, and, more intriguing, the high anti-lymphoma efficacy and the very low relapse rate observed after ASCT.

## Methods

### Patients

From October 2009 to February 2012, all consecutive HIV^+^ patients with NHL who received ASCT as first-line consolidation or as salvage therapy at our Institution were enrolled in this prospective study. Patients signed an informed consent and the project was carried out in agreement with Declaration of Helsinki principles. Approval for these studies was obtained by our Institutional Board of Ethics Committee (Institution: ASST Spedali Civili di Brescia, Brescia, Italy; approved protocol no. NP 2352). Blood samples were obtained from patients at different time points: the day before starting the conditioning regimen (T0), and at 3 (T3), 6 (T6), 12 (T12), and 24 (T24) months after ASCT. Results obtained in HIV^+^ patients were compared with those of HIV^−^ patients and those of age-matched HC. Peripheral blood mononuclear cells (PBMC) were prepared by Ficoll-Hypaque gradient centrifugation, and frozen in liquid nitrogen until use.

### Quantification of lymphocyte subpopulations

Newly produced T and B lymphocytes were quantified by measuring TRECs and KRECs in PBMC using a duplex quantitative real-time PCR performed as previously reported^39^. Results were expressed as copies/mL of blood.

For B-cell subpopulation identification, one million PBMCs from HIV^+^, HIV^−^ and HC were phenotyped after staining with peridin-clorophyll protein-Cy5.5 anti-CD19, phycoerythrin-Cy7 anti-CD10, fluorescein isothiocyanate anti-IgD, and phycoerythrin anti-CD27 mAbs. The cells were first gated for CD19 expression on lymphocytes and then analyzed for the expression of CD10 marker to identify CD19^+^CD10^+^ immature B cells and CD19^+^CD10^−^ mature B cells. This last subset was examined for IgD and CD27 molecule expression in order to recognize IgD^+^CD27^−^ naïve B cells, IgD^+^CD27^+^ unswitched memory B cells, and IgD^−^CD27^+^ switched memory B cells. For T-cell subpopulation characterization, one million PBMCs were stained with phycoerythrin anti-CD3, allophycocyanin-H7 anti-CD4, phycoerythrin-Cy7 anti-CD8, fluorescein isothiocyanate anti-CD45RA, peridin-clorophyll protein-Cy5.5 anti-CCR7, and allophycocyanin anti-CD31 mAbs. PBMCs were first gated on the basis of CD3 expression, analyzed for CD4 and CD8 markers, and then for the expression of CD45RA and CCR7 in order to identify: CD45RA^+^CCR7^+^CD4^+^ and CD45RA^+^CCR7^+^CD8^+^ naïve T lymphocytes; CD45RA^−^CCR7^+^ central memory (T_CM_) and CD45RA^−^CCR7^−^ effector memory (T_EM_). RTE were naïve CD4^+^ T lymphocytes expressing the CD31 molecule. mAbs were purchased from BD Pharmingen, eBioscience, BioLegend (San Diego, CA) and Miltenyi Biotec (Bergisch Gladbach, Germany). Data were analysed with the FACS Diva software (BD Bioscience, San Diego, CA), and reported as absolute counts per μL of blood.

### Average number of *in-vivo* B-cell divisions and *in-vitro* T- and B-cell activation

The replication history of B lymphocytes was evaluated calculating the differences between the cycle threshold numbers (ΔCt) obtained by real-time PCR of the signal joint and the coding joint, which are generated during the rearrangement of IGK genes^40^. For proliferation assay, PBMC (3–5 × 10^6^/mL) prepared using samples obtained at 24 months since ASCT were labelled with 0.2 μmol/L of carboxyfluorescein succinimidyl ester (CFSE; Invitrogen, Eugene, OR) for 20 minutes. Cells were plated in 96-well U-bottom culture plates and stimulated at 37 °C with 6.25 μg/mL phytohemagglutinin or 10 μg/mL CpG ODN 2006 (InvivoGen, San Diego, CA), 5 μg/mL of F(ab)_2_ anti-human IgM/IgG/IgA (Jackson Immunoresearch, West Grove, PA), 40 U/mL interleukin-2 and 50 ng/mL interleukin-10 (Sigma-Aldrich, St Louis, MO), as described elsewhere^23^,^41^. T- and B-cell proliferation was measured by flow cytometry after 5 days of culture. Data were analysed as previously reported^42^.

### TCR repertoire analysis by complementarity-determining region 3 (CDR3) spectratyping

The diversity of TCR beta variable (TCRBV) families was studied by spectratyping after performing multiplex PCR^43^. The length distributions of PCR products were analyzed on an ABI 3500 Genetic Analyzer; distribution of fragment lengths, number of detectable peaks per TCRBV element, and area under the curve were calculated by Gene Mapper (Applied Biosystems). The CDR3 size distribution of TCRBV families of each subject was classified into four categories: normal (Gaussian distribution and >7 peaks), shifted (deviation from Gaussian distribution and >7 peaks), restricted (prominent deviation from Gaussian distribution), and mono/oligoclonal (1 or 2 dominant peaks)^43^. Furthermore, the distribution of TCRBV perturbations was also calculated using the generalized Hamming distance method^44^, in which the CDR3 length distribution of each TCRBV of a patient was subtracted from the average Gaussian-like CDR3 length distribution obtained by analysing a “reference group” composed of age-matched HC. When a TCRBV family was not represented (no detectable peaks), the condition of maximal perturbation was reached, and its value was arbitrarily set to 100%.

### Statistical analysis

Comparisons between the mean of quantitative variables measured in HIV^+^ and HIV^−^ subjects at several time points during the follow-up were performed by repeated measures ANOVA using linear mixed-models with a random slope. In particular, comparisons between the mean values of lymphocyte subpopulation counts, including log-KRECs and log-TRECs, measured in HIV^+^ and HIV^−^ subjects at more than one time point during the follow-up were performed by repeated measures ANOVA using linear mixed-models with a random slope. In these models, HIV serostatus was considered as a covariate and part of an interaction term with time. A similar model was employed to compare immune reconstitution in HIV^+^ patients, using a binary covariate to compare partial vs complete remission. The same technique was used to compare average perturbations (after arcsine data transformation). In this case, the covariate included the HC group. When this interaction was significant, post-hoc comparisons between HIV^+^ and HIV^−^ subjects at the different time points were performed by linear contrasts, and Bonferroni corrected p-values were calculated. On the contrary, if the interaction was not significant, but a main effect with more than two levels was significant, Bonferroni corrected post-hoc comparisons were used to compare the differences between the levels. Alternatively, in case the significant main effect included only two levels, only its p-value was reported (e.g. HIV^+^ vs HIV^−^). In the case of rituximab treatment, only planned contrasts were used to compare the means of the cell population counts between the three subgroups at the different time points. *In-vitro* patient cell proliferations were compared to those of HC by one-way ANOVA followed by the Dunnett’s test. Comparisons between categorical variables were performed by the Fisher’s exact test. Differences were considered significant when P < 0.05.

## Additional Information

**How to cite this article**: Bertoli, D. *et al*. B- and T-lymphocyte number and function in HIV^+^/HIV^−^ lymphoma patients treated with high-dose chemotherapy and autologous bone marrow transplantation. *Sci. Rep.* **6**, 37995; doi: 10.1038/srep37995 (2016).

**Publisher's note:** Springer Nature remains neutral with regard to jurisdictional claims in published maps and institutional affiliations.

## Supplementary Material

Supplementary Data

## Figures and Tables

**Table 1:** Main characteristics and clinical data of HIV^+^ and HIV^−^ patients with non-Hodgkin lymphoma receiving HDC and ASCT.

	HIV^+^ patients (#=11)^a^	HIV^−^ patients (#=9)
	number (%)^b^	number (%)
Sex (males)	11 (100%)	5 (56%)*
Age: median years (range)	46 (29–54)	51 (27–70)
Detectable HIV-viral load at enrolment	1 (9%)^c^	na
Hepatitis B surface antigen positive	0 (0%)	1 (11%)
Hepatitis C virus positive	3 (27%)	0 (0%)
Epstein Barr virus positive	4^d^	1
Histology		
DLBCL	5 (45%)	8 (89%)
Plasmablastic lymphoma	4 (36%)	0 (0%)
Anaplastic large cell lymphoma ALK-negative	1 (9%)	0 (0%)
Intermediate BL/DLBCL	1 (9%)	0 (0%)
High grade B-cell lymphoma, unclassified	0 (0%)	1 (11%)
Ann-Arbor classification		
Stage I–II	1 (9%)	2 (22%)
Stage III–IV	10 (91%)	7 (78%)
Disease status		
1^st^ complete remission	3 (27%)	4 (44%)
2^nd^ complete remission	1 (9%)	1 (11%)
1^st^ partial remission	6 (55%)	4 (44%)
chemosensitive relapse	1 (9%)	0 (0%)
Previous lines of therapies:		
1	7 (64%)	7 (78%)
2	3 (27%)	2 (22%)
3	1 (9%)	0 (0%)
Rituximab treatment before ASCT	5 (45%)	9 (100%)*
Conditioning regimen:		
BEAM^e^	10 (91%)	9 (100%)
Carmustine-thiotepa^f^	1 (9%)	0 (0%)
T0^g^ versus the last day of induction chemotherapy: median days (range)	55 (40–212)	50 (36–132)
T0^g^ versus the last rituximab administration: median days (range)	98 (43–107)	73 (43–139)
Mobilized CD34^+^cells: median × 10^6^ (range)	5.8 (2.65–12.9)	16.3 (6.4–20.7)*
Infused CD34^+^cells: median × 10^6^/Kg of body weight (range)	5.8 (2.65–9.5)	6.9 (4.4–9.4)
Neutrophil engraftment: median days (range)	10 (9–12)	9 (8–11)
Late onset neutropenia	2 (18%)	2 (22%)
Platelet engraftment: median days (range)	13 (10–16)	12 (10–16)
Radiotherapy after ASCT	6 (55%)	3 (33%)

Abbreviations: na, not applicable; ns, not significant; ALK, anaplastic lymphoma kinase; DLBCL, diffuse large B-cell lymphoma; BL, Burkitt lymphoma.

*P < *0.05* (P-value calculations were done by the Fisher exact test for categorical variables and unpaired t test for continuous variables).

^a^Three patients were intravenous drug users, 4 were men who had sex with men and 4 were heterosexual. Two patients had an AIDS-defining condition other than NHL: 1 cutaneous Kaposi sarcoma and 1 extrapulmonary tuberculosis;

^b^unit of measure is count (percentage) unless differently specified;

^c^viremia: 2.374 copies/mL;

^d^EBV was detected by using immunohistochemistry only in 4 out 7 HIV^+^ and in 1 out 3 HIV^−^ patients analysed;

^e^carmustine 300 mg/m^2^, cytarabine 800 mg/m^2^, etoposide 800 mg/m^2^, and melphalan 140 mg/m^2^;

^f^carmustine 400 mg/m^2^, thiotepa 20 mg/Kg;

^g^T0: the day preceding the conditioning regimen.
