# Supplementary material for: B- and T-lymphocyte number and function in HIV+/HIV− lymphoma patients treated with high-dose chemotherapy and autologous bone marrow transplantation
Source: Sci Rep. 2016 Dec 1;6:37995. doi: 10.1038/srep37995 (PMC5131356; doi:10.1038/srep37995)

B- and T-lymphocyte number and function in HIV$^+$/HIV$^-$ lymphoma patients treated with high-dose chemotherapy and autologous bone marrow transplantation

Diego Bertoli[1], Alessandro Re[2], Marco Chiarini[1], Alessandra Sottini[1], Federico Serana[1], Viviana Giustini[1], Aldo M. Roccaro[1], Chiara Cattaneo[2], Luigi Caimi[1], Giuseppe Rossi[2], Luisa Imberti[1*]
[1]Centro di Ricerca Emato-oncologica AIL (CREA), ASST Spedali Civili, Brescia, Italy
[2]Hematology, ASST Spedali Civili, Brescia, Italy

SUPPLEMENTARY FIGURES

Supplementary Figure 1. Number of memory CD4$^+$ and CD8$^+$ lymphocyte subsets.
CD4$^+$ and CD8$^+$ lymphocyte subsets were evaluated at the indicated time points in HIV$^+$ (black dots) and HIV$^-$ (white squares) patients. Central memory cells (TCM) are CD45RA$^-$CCR7$^+$ (A)(C), effector memory (TEM) are CD45RA$^-$CCR7$^-$ (B)(D). Error bars represent the 95% confidence interval of the mean. Grey horizontal lines represents the reference range defined as the highest and lowest values found in HC. Arrows made by both dashed and solid lines indicate significant differences between time-point and T0, independent of patient group (i.e. interaction non-significant). HC: healthy controls; T0: before ASCT (baseline), T3, T6, T12, and T24: at 3, 6, 12, and 24 months after ASCT, respectively.

Supplementary Figure 2. TCR repertoire changes.
(A) Representation of TCR repertoire changes at the single single-TCRBV chain, single-patient level. Bars represent the difference in perturbations between T24 and T0 (red bars: positive difference i.e. increased perturbations, blue bars: negative difference). The average difference in TCRBV perturbations between T24 and T0 at the single-patient level was analyzed by the Wilcoxon signed rank test. (B) Dendrogram showing an unsupervised hierarchical clustering of patients (obtained using the dCHIP software, http://www.dchip.org, and based on standardized perturbation fold-change between T24 and T0), which allowed us to classify patients as shown by the band on top: all patients but one per each group (HIV$^+$, red; HIV$^-$ patients, green) were correctly classified within the two clusters.

SUPPLEMENTARY FIGURE 1

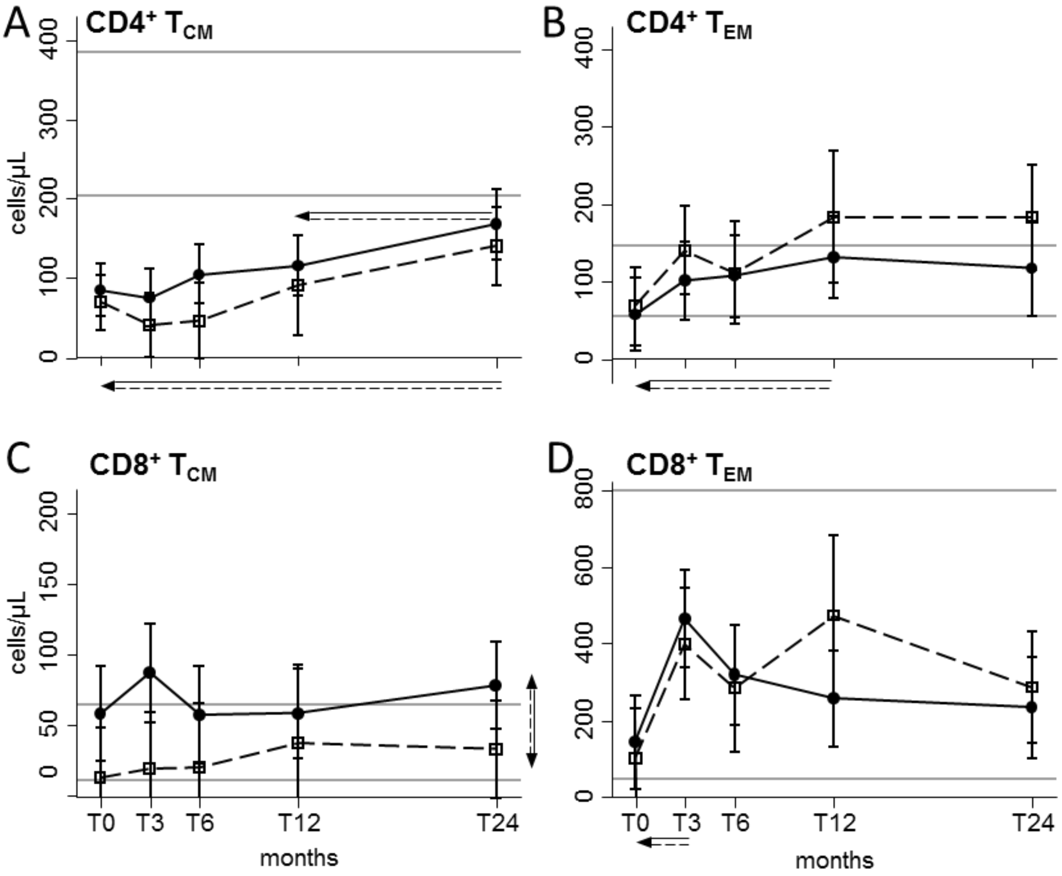

SUPPLEMENTARY FIGURE 2

A

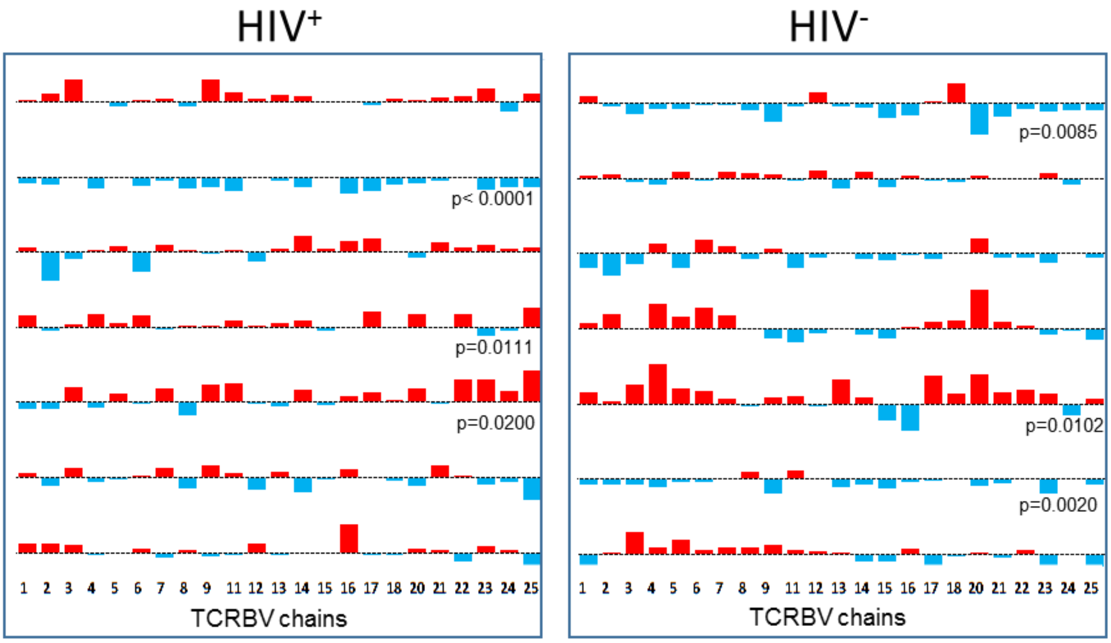

B

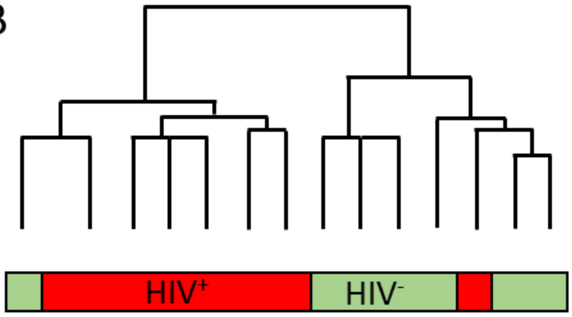

Supplement: Supplementary Data [file srep37995-s1.pdf]
